# Supplementary material for: Sex-Specific HLA Alleles Contribute to the Modulation of COVID-19 Severity
Source: Int J Mol Sci. 2024 Dec 8;25(23):13198. doi: 10.3390/ijms252313198 (PMC11642212; doi:10.3390/ijms252313198)
Supplement: Supplementary file 1 [file ijms-25-13198-s001.zip › ijms-3255190-table s1.pdf]

HLA-locus	
**HLA-A**	NM_001126112.3
**HLA-B**	NM_004124.3
**HLA-C**	NM_001197.3
**HLA-DMA**	NM_006120.4
**HLA-DMB**	NM_002118.5
**HLA-DOA**	NM_001130539.1
**HLA-DOB**	NM_001130540.1
**HLA-DPA1**	NM_001130541.1
**HLA-DPB1**	NM_002121.6
**HLA-DQA1**	NM_033554.4
**HLA-DQA2**	NM_020056.5
**HLA-DQB1**	NM_001243962.1
**HLA-DQB2**	NM_001300790.2
**HLA-DRA**	NM_019111.5
**HLA-E**	NM_005516.6
**HLA-F**	NM_005516.3
**HLA-G**	NM_001384280.1

Supplementary Table1: HLA genes sequenced in the study and relative NM_id.

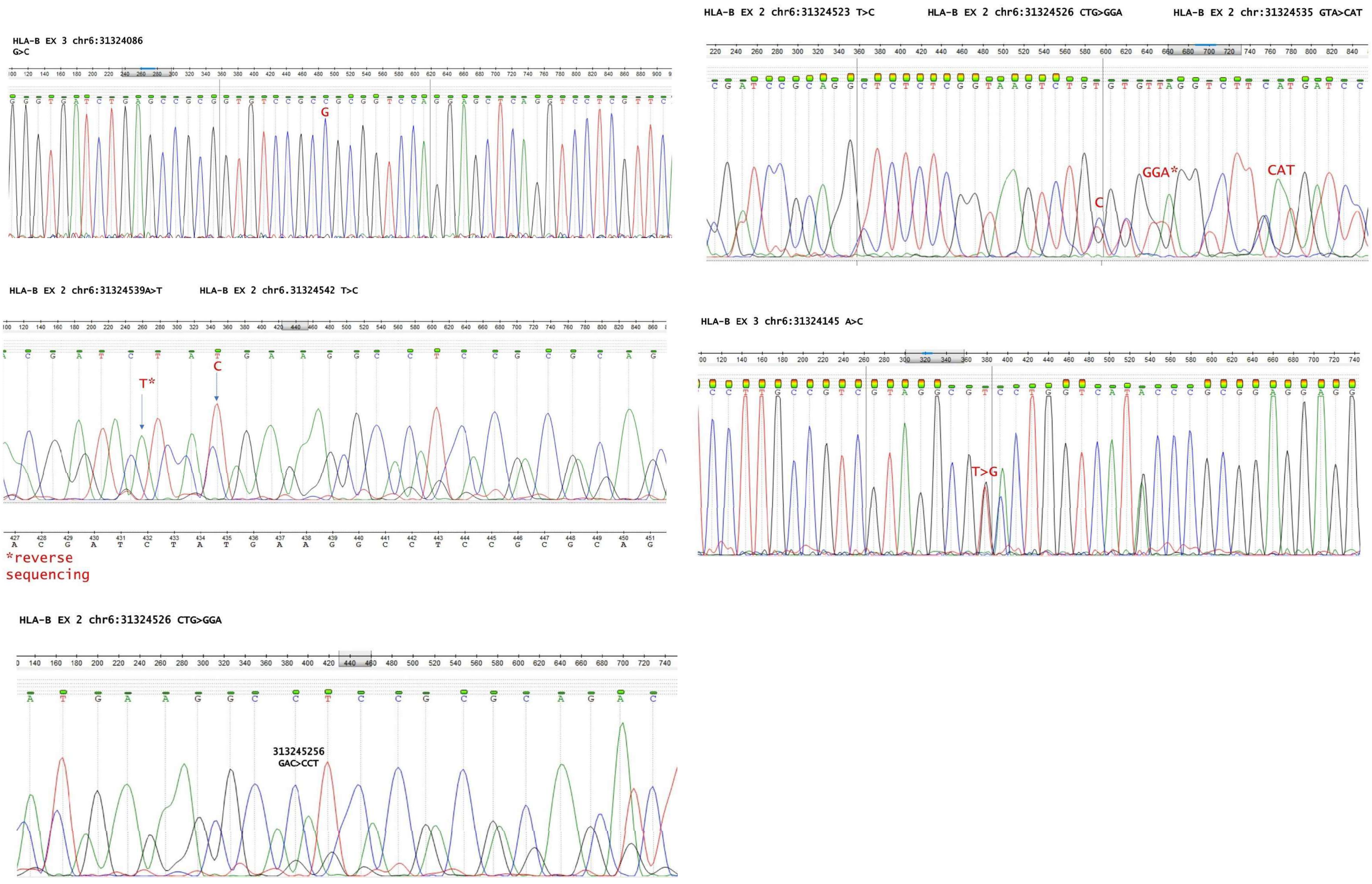

Supplementary Fig 1a: Sanger Sequencing confirmation of significative variants of *HLA-B* gene.

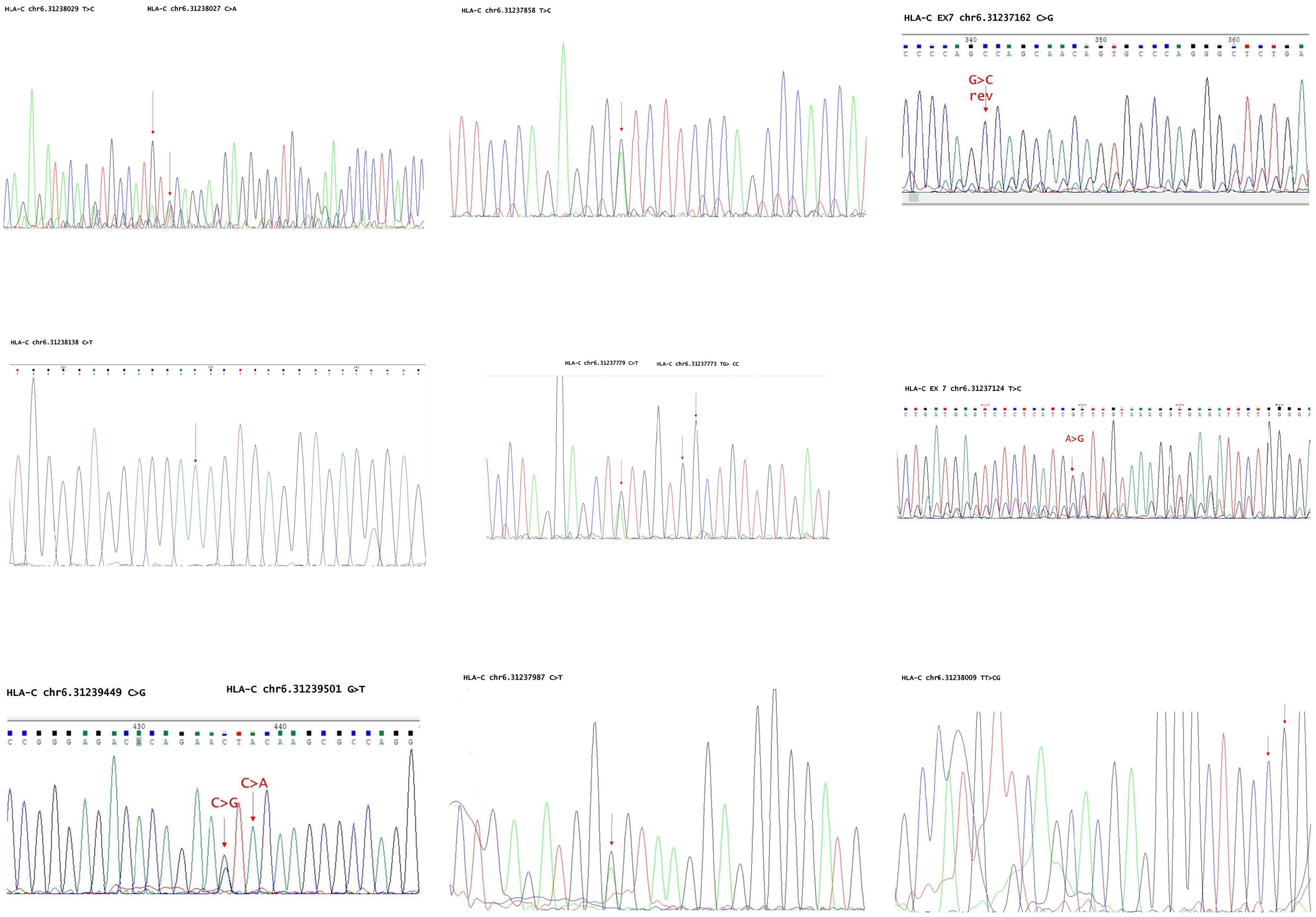

Supplementary Fig 1b: Sanger Sequencing confirmation of significant variants of the HLA-C gene.

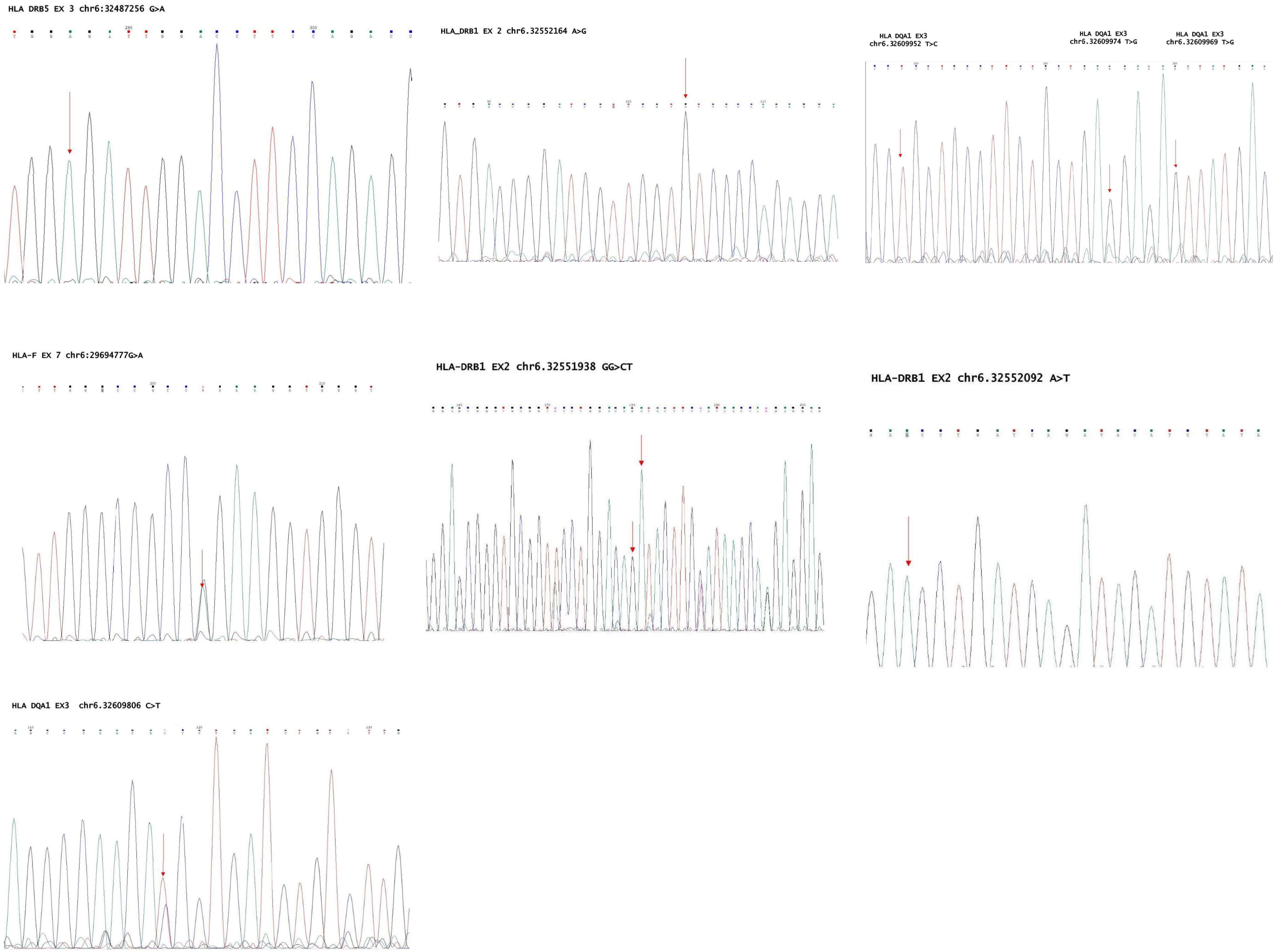

Supplementary Fig 1c: Sanger Sequencing confirmation of variants in HLA-*DRB5*,-*DRB1*,-*DQA1*, AND -*F.*

Haplotype ID #	gDNA (chr6:)	31324542T>C	31324539A>T	31324535GTA>CAT	31324526CTG>GGA	31324523T>C	31324145A>C	31324086G>C	aminoacidic change						
	cDNA	266A>G	269T>A	271TAC>ATG	280CAG>TCC	285A>G	418T>G	477C>G	Gln89Arg	Ile90Thr	Tyr91Met	Gln94Ser	Ala95=	Tyr140Asp	Ala159=
1		G	A	ATG	CAG	A	T	C	Arg	Thr	Met	Gln	Ala	Tyr	Ala
2		A	T	TAC	TCC	G	T	G	Gln	Ile	Tyr	Ser	Ala	Tyr	Ala
3		A	T	TAC	CAG	A	T	C	Gln	Ile	Tyr	Gln	Ala	Tyr	Ala
4		G	A	ATG	TCC	G	T	C	Arg	Thr	Met	Ser	Ala	Tyr	Ala
5		A	T	TAC	CAG	A	T	G	Gln	Ile	Tyr	Gln	Ala	Tyr	Ala
6		G	A	ATG	TCC	G	T	G	Arg	Thr	Met	Ser	Ala	Tyr	Ala
7		A	T	TAC	TCC	A	T	C	Gln	Ile	Tyr	Ser	Ala	Tyr	Ala
8		G	T	ATG	TCC	A	T	G	Arg	Ile	Met	Ser	Ala	Tyr	Ala
9		A	T	TAC	CAG	A	G	C	Gln	Ile	Tyr	Gln	Ala	Asp	Ala
10		G	A	ATG	CAG	G	T	C	Arg	Thr	Met	Gln	Ala	Tyr	Ala
11		A	T	TAC	TCC	A	T	G	Gln	Ile	Tyr	Ser	Ala	Tyr	Ala
12		G	A	ATG	TCC	G	G	G	Arg	Thr	Met	Ser	Ala	Asp	Ala
13		G	A	ATG	TCC	G	G	C	Arg	Thr	Met	Ser	Ala	Asp	Ala
14		A	T	ATG	TCC	A	T	G	Gln	Ile	Met	Ser	Ala	Tyr	Ala
15		A	T	TAC	CAG	A	G	G	Gln	Ile	Tyr	Gln	Ala	Asp	Ala
16		G	A	ATG	CAG	G	G	G	Arg	Thr	Met	Gln	Ala	Asp	Ala
17		A	T	ATG	TCC	A	T	C	Gln	Ile	Met	Ser	Ala	Tyr	Ala

Supplementary Table 2a: Haplotypes inferred in *HLA-B* gene.

Haplotype ID#	gDNA (chr6:)	31239501.G.T	31239449.C.G	31238138.C.T	31238029.T.C	31238027.C.A	31238009.TT.CG	31237987.C.T	31237858.T.C	31237779.C.T	31237774.G.C	31237773.T.C	31237162.C.G	31237124.T.C	Aminoacidic change											
	cDNA	218C>A	270G>C	744G>A	853A>G	855G>T	872AA>CG	895G>A	900A>G	979G>A	984C>G	985A>G	1049G>C	1087A>G	Ala73Glu	Lys90Asn	Gln248His	Met285Val	Gln291Pro	Glu299*	Pro300=	Val327Leu	Val328=	Thr329Ala	Cys350Ser	Thr363Ser
1		A	G	A	G	T	CG	A	G	A	G	G	C	G	Glu	Lys	His	Val	Pro	*	Pro	Leu	Val	Ala	Ser	Ser
2		C	G	G	A	G	CG	A	G	G	G	G	C	G	Ala	Lys	Gln	Met	Pro	*	Pro	Val	Val	Ala	Ser	Ser
3		C	G	A	G	G	CG	G	G	G	G	G	C	G	Ala	Lys	His	Val	Pro	Glu	Pro	Val	Val	Ala	Ser	Ser
4		C	C	G	A	G	AA	G	A	G	C	A	G	A	Ala	Asn	Gln	Met	Gln	Glu	Pro	Val	Val	Thr	Cys	Thr
5		A	G	G	G	T	CG	A	A	A	G	G	C	G	Glu	Lys	Gln	Val	Pro	*	Pro	Leu	Val	Ala	Ser	Ser
6		C	G	A	G	G	CG	G	A	G	G	G	C	G	Ala	Lys	His	Val	Pro	Glu	Pro	Val	Val	Ala	Ser	Ser
7		C	C	G	A	G	AA	G	A	G	G	G	C	G	Ala	Asn	Gln	Met	Gln	Glu	Pro	Val	Val	Ala	Ser	Ser
8		C	G	G	A	G	AA	G	A	G	G	G	C	G	Ala	Lys	Gln	Met	Gln	Glu	Pro	Val	Val	Ala	Ser	Ser
9		C	G	G	A	G	CG	G	A	G	G	G	C	G	Ala	Lys	Gln	Met	Pro	Glu	Pro	Val	Val	Ala	Ser	Ser
10		C	G	G	G	G	AA	G	A	G	G	G	C	G	Ala	Lys	Gln	Val	Gln	Glu	Pro	Val	Val	Ala	Ser	Ser
11		C	G	G	A	G	AA	G	A	G	C	A	G	A	Ala	Lys	Gln	Met	Gln	Glu	Pro	Val	Val	Thr	Cys	Thr
12		C	G	G	A	G	CG	G	G	G	G	G	C	G	Ala	Lys	Gln	Met	Pro	Glu	Pro	Val	Val	Ala	Ser	Ser
13		A	G	G	G	T	CG	G	A	A	G	G	C	G	Glu	Lys	Gln	Val	Pro	Glu	Pro	Leu	Val	Ala	Ser	Ser
14		A	G	G	G	T	CG	A	G	A	G	G	C	G	Glu	Lys	Gln	Val	Pro	*	Pro	Leu	Val	Ala	Ser	Ser
15		C	G	A	G	G	CG	G	G	G	C	A	G	A	Ala	Lys	His	Val	Pro	Glu	Pro	Val	Val	Thr	Cys	Thr
16		C	C	G	A	G	CG	G	A	G	G	G	C	G	Ala	Asn	Gln	Met	Pro	Glu	Pro	Val	Val	Ala	Ser	Ser
17		C	G	A	G	G	AA	G	A	G	G	G	C	G	Ala	Lys	His	Val	Gln	Glu	Pro	Val	Val	Ala	Ser	Ser
18		A	G	G	G	T	CG	G	A	A	G	G	C	A	Glu	Lys	Gln	Val	Pro	Glu	Pro	Leu	Val	Ala	Ser	Thr
19		C	C	A	G	G	CG	G	A	G	G	G	C	G	Ala	Asn	His	Val	Pro	Glu	Pro	Val	Val	Ala	Ser	Ser
20		A	G	A	G	T	CG	A	G	A	C	A	G	A	Glu	Lys	His	Val	Pro	*	Pro	Leu	Val	Thr	Cys	Thr
21		C	C	G	G	G	CG	G	A	G	G	G	C	G	Ala	Asn	Gln	Val	Pro	Glu	Pro	Val	Val	Ala	Ser	Ser
22		C	G	G	A	G	AA	G	A	G	G	G	G	G	Ala	Lys	Gln	Met	Gln	Glu	Pro	Val	Val	Ala	Cys	Ser
23		C	G	G	A	G	CG	G	A	G	C	A	G	A	Ala	Lys	Gln	Met	Pro	Glu	Pro	Val	Val	Thr	Cys	Thr
24		C	G	G	G	G	CG	G	A	G	G	G	C	G	Ala	Lys	Gln	Val	Pro	Glu	Pro	Val	Val	Ala	Ser	Ser
25		C	C	G	A	G	CG	G	A	G	C	A	G	A	Ala	Asn	Gln	Met	Pro	Glu	Pro	Val	Val	Thr	Cys	Thr
26		C	G	A	G	G	AA	G	A	G	G	G	C	A	Ala	Lys	His	Val	Gln	Glu	Pro	Val	Val	Ala	Ser	Thr
27		C	G	G	A	G	AA	G	A	G	C	A	C	G	Ala	Lys	Gln	Met	Gln	Glu	Pro	Val	Val	Thr	Ser	Ser
28		C	G	A	G	G	CG	A	G	G	G	G	C	G	Ala	Lys	His	Val	Pro	*	Pro	Val	Val	Ala	Ser	Ser
29		C	C	G	A	G	CG	G	A	G	C	A	C	G	Ala	Asn	Gln	Met	Pro	Glu	Pro	Val	Val	Thr	Ser	Ser
30		C	G	G	G	G	AA	G	A	G	C	A	G	G	Ala	Lys	Gln	Val	Gln	Glu	Pro	Val	Val	Thr	Cys	Ser
31		C	G	G	A	G	CG	G	A	G	G	G	G	G	Ala	Lys	Gln	Met	Pro	Glu	Pro	Val	Val	Ala	Cys	Ser
32		C	G	G	A	G	CG	G	A	G	C	A	C	G	Ala	Lys	Gln	Met	Pro	Glu	Pro	Val	Val	Thr	Ser	Ser
33		C	G	G	G	G	AA	G	A	G	G	G	C	A	Ala	Lys	Gln	Val	Gln	Glu	Pro	Val	Val	Ala	Ser	Thr
34		C	G	A	G	G	AA	A	G	G	C	A	C	G	Ala	Lys	His	Val	Gln	*	Pro	Val	Val	Thr	Ser	Ser
35		A	G	A	G	T	CG	A	G	A	G	G	G	G	Glu	Lys	His	Val	Pro	*	Pro	Leu	Val	Ala	Cys	Ser
36		C	C	G	A	G	AA	G	A	G	C	A	G	G	Ala	Asn	Gln	Met	Gln	Glu	Pro	Val	Val	Thr	Cys	Ser
37		A	G	G	G	T	CG	G	G	A	G	G	C	G	Glu	Lys	Gln	Val	Pro	Glu	Pro	Leu	Val	Ala	Ser	Ser
38		C	G	G	A	G	AA	G	A	G	G	G	G	A	Ala	Lys	Gln	Met	Gln	Glu	Pro	Val	Val	Ala	Cys	Thr
39		C	G	A	A	G	AA	G	A	G	G	G	C	A	Ala	Lys	His	Met	Gln	Glu	Pro	Val	Val	Ala	Ser	Thr
40		C	C	G	A	G	AA	G	A	G	G	G	G	A	Ala	Asn	Gln	Met	Gln	Glu	Pro	Val	Val	Ala	Cys	Thr
41		C	G	A	G	G	AA	G	A	A	G	G	C	A	Ala	Lys	His	Val	Gln	Glu	Pro	Leu	Val	Ala	Ser	Thr
42		C	G	G	G	G	CG	G	A	G	G	G	C	A	Ala	Lys	Gln	Val	Pro	Glu	Pro	Val	Val	Ala	Ser	Thr
43		C	G	G	G	G	CG	G	G	G	G	G	C	G	Ala	Lys	Gln	Val	Pro	Glu	Pro	Val	Val	Ala	Ser	Ser
44		C	G	G	A	G	AA	G	A	G	G	G	C	A	Ala	Lys	Gln	Met	Gln	Glu	Pro	Val	Val	Ala	Ser	Thr
45		C	G	G	G	T	CG	A	G	A	G	G	C	G	Ala	Lys	Gln	Val	Pro	*	Pro	Leu	Val	Ala	Ser	Ser
46		C	G	G	G	T	CG	A	G	G	G	G	G	G	Ala	Lys	Gln	Val	Pro	*	Pro	Val	Val	Ala	Cys	Ser
47		C	G	A	G	T	CG	A	G	A	G	G	C	G	Ala	Lys	His	Val	Pro	*	Pro	Leu	Val	Ala	Ser	Ser
48		C	G	A	A	G	AA	G	A	A	G	G	C	A	Ala	Lys	His	Met	Gln	Glu	Pro	Leu	Val	Ala	Ser	Thr
49		C	G	A	G	G	AA	G	A	A	G	G	G	A	Ala	Lys	His	Val	Gln	Glu	Pro	Leu	Val	Ala	Cys	Thr
50		C	G	G	G	G	CG	G	A	G	G	G	G	A	Ala	Lys	Gln	Val	Pro	Glu	Pro	Val	Val	Ala	Cys	Thr
51		C	G	G	G	T	CG	G	A	A	G	G	C	G	Ala	Lys	Gln	Val	Pro	Glu	Pro	Leu	Val	Ala	Ser	Ser
52		C	G	A	G	T	CG	A	A	A	G	G	G	G	Ala	Lys	His	Val	Pro	*	Pro	Leu	Val	Ala	Cys	Ser
53		A	G	A	G	T	CG	A	A	A	G	G	C	G	Glu	Lys	His	Val	Pro	*	Pro	Leu	Val	Ala	Ser	Ser
54		C	G	G	A	G	AA	A	A	G	C	A	G	G	Ala	Lys	Gln	Met	Gln	*	Pro	Val	Val	Thr	Cys	Ser
55		A	G	A	G	T	CG	A	A	A	C	A	G	A	Glu	Lys	His	Val	Pro	*	Pro	Leu	Val	Thr	Cys	Thr
56		C	C	G	A	G	AA	G	A	G	C	A	C	G	Ala	Asn	Gln	Met	Gln	Glu	Pro	Val	Val	Thr	Ser	Ser
57		C	G	G	G	G	CG	G	A	G	C	A	C	G	Ala	Lys	Gln	Val	Pro	Glu	Pro	Val	Val	Thr	Ser	Ser
58		C	G	G	G	G	AA	G	A	G	C	A	C	G	Ala	Lys	Gln	Val	Gln	Glu	Pro	Val	Val	Thr	Ser	Ser
59		C	G	A	G	G	CG	G	A	G	G	G	C	A	Ala	Lys	His	Val	Pro	Glu	Pro	Val	Val	Ala	Ser	Thr
60		A	G	G	G	T	CG	A	G	G	C	A	C	G	Glu	Lys	Gln	Val	Pro	*	Pro	Val	Val	Thr	Ser	Ser
61		A	G	G	A	G	CG	G	A	A	G	G	C	G	Glu	Lys	Gln	Met	Pro	Glu	Pro	Leu	Val	Ala	Ser	Ser
62		A	G	A	G	T	CG	A	G	A	C	A	C	G	Glu	Lys	His	Val	Pro	*	Pro	Leu	Val	Thr	Ser	Ser
63		A	G	A	G	T	CG	A	G	A	C	A	C	A	Glu	Lys	His	Val	Pro	*	Pro	Leu	Val	Thr	Ser	Thr
64		A	G	G	G	T	CG	G	G	G	G	G	C	G	Glu	Lys	Gln	Val	Pro	Glu	Pro	Val	Val	Ala	Ser	Ser
65		C	G	A	G	G	AA	G	A	G	C	A	C	G	Ala	Lys	His	Val	Gln	Glu	Pro	Val	Val	Thr	Ser	Ser
66		C	G	G	A	G	AA	G	G	G	C	A	C	G	Ala	Lys	Gln	Met	Gln	Glu	Pro	Val	Val	Thr	Ser	Ser
67		A	G	A	A	G	AA	A	A	A	G	G	C	G	Glu	Lys	His	Met	Gln	*	Pro	Leu	Val	Ala	Ser	Ser
68		C	G	G	A	G	AA	A	G	G	C	A	C	G	Ala	Lys	Gln	Met	Gln	*	Pro	Val	Val	Thr	Ser	Ser
69		A	G	A	A	G	AA	G	A	A	G	G	C	G	Glu	Lys	His	Met	Gln	Glu	Pro	Leu	Val	Ala	Ser	Ser
70		C	C	G	G	G	AA	G	A	G	G	G	C	G	Ala	Asn	Gln	Val	Gln	Glu	Pro	Val	Val	Ala	Ser	Ser
71		C	G	A	G	G	AA	A	A	G	C	A	C	G	Ala	Lys	His	Val	Gln	*	Pro	Val	Val	Thr	Ser	Ser
72		C	G	G	G	T	CG	A	A	A	G	G	C	G	Ala	Lys	Gln	Val	Pro	*	Pro	Leu	Val	Ala	Ser	Ser
73		C	C	G	G	G	CG	G	A	G	G	G	G	G	Ala	Asn	Gln	Val	Pro	Glu	Pro	Val	Val	Ala	Cys	Ser
74		C	G	G	G	T	CG	G	G	G	G	G	C	G	Ala	Lys	Gln	Val	Pro	Glu	Pro	Val	Val	Ala	Ser	Ser
75		C	G	A	G	G	CG	G	G	G	C	A	C	G	Ala	Lys	His	Val	Pro	Glu	Pro	Val	Val	Thr	Ser	Ser
76		C	C	A	A	G	AA	G	A	G	G	G	C	G	Ala	Asn	His	Met	Gln	Glu	Pro	Val	Val	Ala	Ser	Ser
77		A	G	A	A	G	AA	G	A	G	C	A	C	G	Glu	Lys	His	Met	Gln	Glu	Pro	Val	Val	Thr	Ser	Ser
78		C	G	A	A	G	AA	G	A	G	G	G	C	G	Ala	Lys	His	Met	Gln	Glu	Pro	Val	Val	Ala	Ser	Ser
79		C	G	A	A	G	AA	G	G	G	G	G	C	G	Ala	Lys	His	Met	Gln	Glu	Pro	Val	Val	Ala	Ser	Ser
80		A	G	A	A	G	AA	G	G	A	G	G	C	G	Glu	Lys	His	Met	Gln	Glu	Pro	Leu	Val	Ala	Ser	Ser
81		C	G	A	A	G	AA	G	A	G	C	A	C	G	Ala	Lys	His	Met	Gln	Glu	Pro	Val	Val	Thr	Ser	Ser
82		A	G	A	A	G	AA	G	A	G	G	G	C	G	Glu	Lys	His	Met	Gln	Glu	Pro	Val	Val	Ala	Ser	Ser
83		C	C	G	G	G	AA	G	A	G	C	A	C	G	Ala	Asn	Gln	Val	Gln	Glu	Pro	Val	Val	Thr	Ser	Ser
84		A	G	G	A	G	AA	G	A	G	C	A	C	G	Glu	Lys	Gln	Met	Gln	Glu	Pro	Val	Val	Thr	Ser	Ser
85		A	G	A	G	G	AA	G	A	G	G	G	C	G	Glu	Lys	His	Val	Gln	Glu	Pro	Val	Val	Ala	Ser	Ser
86		A	G	A	G	G	AA	G	A	G	C	A	C	G	Glu	Lys	His	Val	Gln	Glu	Pro	Val	Val	Thr	Ser	Ser
87		A	G	A	G	G	AA	G	A	A	G	G	C	G	Glu	Lys	His	Val	Gln	Glu	Pro	Leu	Val	Ala	Ser	Ser
88		C	C	A	G	G	AA	G	A	G	G	G	C	G	Ala	Asn	His	Val	Gln	Glu	Pro	Val	Val	Ala	Ser	Ser
89		C	C	A	A	G	AA	G	A	G	C	A	C	G	Ala	Asn	His	Met	Gln	Glu	Pro	Val	Val	Thr	Ser	Ser

Supplementary Table 2b: Haplotypes inferred in *HLA-C* gene.

gene

DQA1	Haplotype ID #	gDNA (chr6:)	32609806C>T	32609952T>C	32609969T>G	32609974T>G	aminoacidic change			
		cDNA	388C>T	534T>C	551T>G	556T>G	Thr130Leu	Phe179Ile	Asp184Glu	Ile186Ser
	1		C	T	T	T	Thr	Phe	Asp	Ile
	2		C	C	G	G	Thr	Leu	Glu	Ser
	3		T	C	G	G	Ile	Leu	Glu	Ser
	4		T	T	T	T	Ile	Phe	Asp	Ile
	5		C	C	T	T	Thr	Leu	Asp	Ile
	6		C	C	G	T	Thr	Leu	Glu	Ile
	7		C	T	T	G	Thr	Phe	Asp	Ser
	8		C	T	G	G	Thr	Phe	Glu	Ser
	9		C	T	G	T	Thr	Phe	Glu	Ile

DRB1	Haplotype ID #	gDNA (chr6:)	32552092A>T	32551939GG>AT	aminoacidic change	
		cDNA	174T>A	317CC>AT	Phe55Tyr	Thr106Asn
	1		T	C	Phe	Thr
	2		T	C	Phe	Thr
	3		T	A	Phe	Asn
	4		A	C	Tyr	Thr
	5		A	A	Tyr	Asn

DRB5	Haplotype ID #	gDNA (chr6:)	32487256G>A	aminoacidic change
		cDNA	543C>T	Asp181=
	1		C	Asp
	2		T	Asp

F	Haplotype ID #	gDNA (chr6:)	29694777G>A	aminoacidic change
		cDNA	1153G>A	Arg385Gln
	1		G	Arg
	2		A	Gln

Supplementary Table 2c: Haplotypes inferred in *HLA-DRB1,-DRB5,-DQA1,-F* gene.
